# Formation of Toxic Oligomeric Assemblies of RNA-binding Protein: Musashi in Alzheimer’s disease

**DOI:** 10.1186/s40478-018-0615-0

**Published:** 2018-10-26

**Authors:** Urmi Sengupta, Mauro Montalbano, Salome McAllen, Gerard Minuesa, Michael Kharas, Rakez Kayed

**Affiliations:** 1 Mitchell Center for Neurodegenerative Diseases, University of Texas Medical Branch, Medical Research Building Room 10.138C , 301 University Blvd, Galveston, TX 77555-1045 USA; 2 Departments of Neurology, Neuroscience and Cell Biology, University of Texas Medical Branch, Galveston, TX 77555 USA; 3 Memorial Sloan-Kettering Cancer Center, New York, 10065 USA

**Keywords:** Musashi proteins, Oligomers, Tau, Alzheimer’s disease

## Abstract

Alzheimer’s disease (AD) is the most common neurodegenerative disorder associated with structural and functional alterations of brain cells causing progressive deterioration of memory and other cognitive functions. Recent studies demonstrate that several neurodegenerative diseases, including AD exhibit RNA-binding proteins (RBPs) pathologies, including TAR DNA -binding protein (TDP-43), fused in sarcoma (FUS), superoxide dismutase (SOD1) and T-interacting antigen-1 (TIA-1), highlighting the role of RBPs in neurodegeneration. One such group of RBPs, Musashi proteins comprised of MSI1 and MSI2, has been long studied in neurogenesis and cancer biology. Herein, we have investigated the aggregation properties of MSI1 and MSI2 by in vitro assays, their expression and accumulation as well as their possible interactions with other cellular proteins, such as tau in AD pathology. We have performed atomic force microscopy, Western blot, and immunoprecipitation to demonstrate the aggregation properties of recombinant Musashi proteins. Furthermore, we have studied cortical brain sections from AD (*N* = 4) and age-matched non-demented subjects (*N* = 4) by Western blot and immunofluorescence microscopy to investigate MSI1 and MSI2 levels and their localization in human brain tissues. Musashi proteins showed in vitro aggregation properties by forming oligomers. We have observed an increase in Musashi proteins levels in AD brain tissues as compared with age-matched non-demented subjects. Moreover, Musashi proteins are observed to form oligomers in the diseased brain tissues. Interestingly, the co-immunofluorescence study has revealed a change in fluorescence pattern of oligomeric Musashi proteins and tau with a high association in the perinuclear area of the cells suggesting changes in function of Musashi proteins. Our data have demonstrated for the first time that MSI1 and MSI2 are present in an oligomeric state in AD brains compared to the age-matched non-demented subjects and that these large assemblies co-localize with tau contributing to the neurodegenerative pathogenesis.

**Electronic supplementary material:**

The online version of this article (10.1186/s40478-018-0615-0) contains supplementary material, which is available to authorized users.

## Introduction

**Fig. 1:**
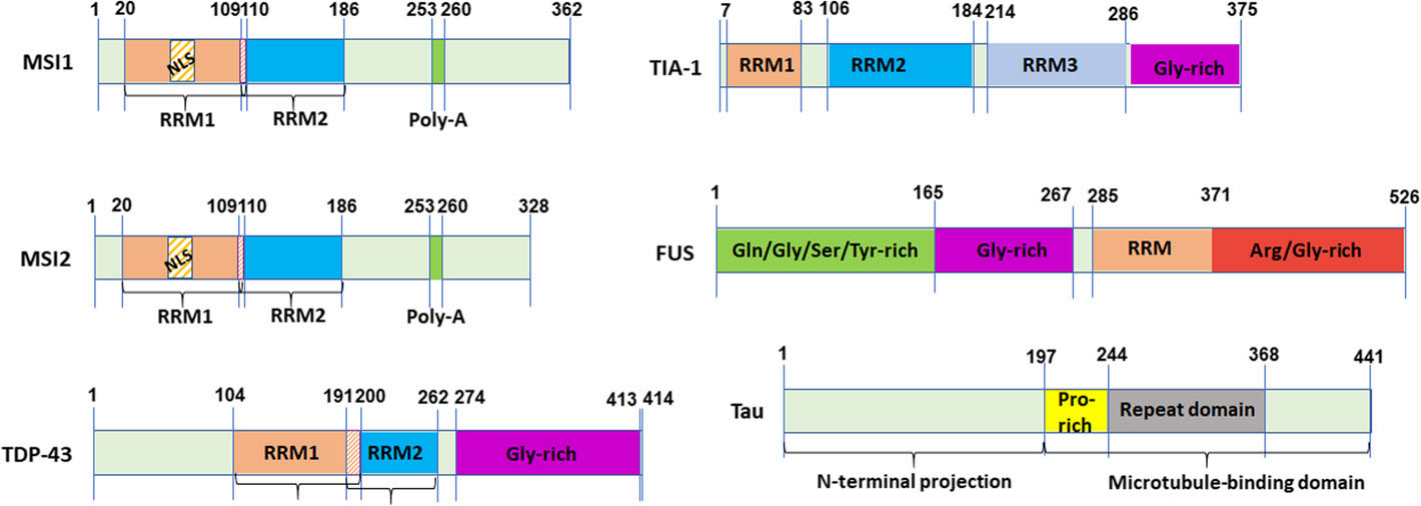
Domain structures of Musashi proteins, alongside TDP-43, FUS and TIA-1, three extensively studied RBPs forming inclusions in neurodegenerative diseases. Tau domain structure is also represented. *NLS* Nuclear Localization Signal

Alzheimer’s disease (AD) is the most common neurodegenerative disorder associated with structural and functional alterations of brain cells causing progressive deterioration of memory and other cognitive functions. Recent studies demonstrate that several neurodegenerative diseases, including AD, exhibit RNA-binding proteins (RBPs) pathologies, such as TAR DNA- binding protein (TDP-43), fused in sarcoma (FUS), superoxide dismutase 1 (SOD1) and T-interacting antigen-1 (TIA-1), highlighting the role of RBPs in neurodegeneration [12, 34, 40]. Musashi proteins belong to a group of RBPs that regulate the translation of target mRNAs during neuronal development, originally observed to regulate asymmetric stem cell division in *Drosophila melanogaster* [42]. The role of this group of proteins is crucial to maintain the pool of adult neuronal stem cells in mammals [45]. They appear to function as translational repressors of target mRNAs encoding cell cycle inhibitory proteins, thus permitting stem cells to maintain an undifferentiated state. Pathological up-regulation of Musashi proteins has been observed in cellular transformation by repressing target mRNAs involved in the inhibition of cell proliferation, as reported in a variety of tumor cells [46], including cancer of neuronal origin [21, 50, 54]. Although the role of Musashi proteins in mRNAs regulation is clearly established, their precise subcellular location is still unclear [43]. In mammals, the two Musashi proteins: MSI1 and MSI2, are composed of 362 and 328 amino acid residues, respectively. Both MSI1 and MSI2 have two RNA-recognition motifs, RRM1 and RRM2. The RRM1 of MSI1 protein contains 20–110 amino acid residues and RRM2 contains 109–186 amino acid residues with a poly-alanine stretch of 274–281 amino acid residues. The RRM1 and RRM2 of MSI2 contain 21–111 amino acid residues and 110–187 amino acid residues with a poly-alanine stretch of 253–260 residues (Fig. 1) [30]. MSI1 is found in both cytoplasm and nucleus, whereas, MSI2 is reported to be associated with the polysomes in the cytoplasm [25, 44]. These proteins are mostly diffused throughout the cytoplasm, but can be nuclear or localized in perinuclear region as well depending on cell types [37]. The mechanisms regulating nuclear localization of Musashi proteins during differentiation are not determined yet [37]. It is still unclear if the nuclear sequestration of Musashi facilitates cytoplasmic target mRNA translation or if MSI1 and − 2 have distinct nuclear functions. Both Musashi proteins are involved in the process of maturation of exon 10+ tau transcripts in neuronal cell lines, indicating potential roles in alternative splicing of certain pre-mRNAs [14]. Among the two paralogs, the functional aspect of MSI1 protein is more extensively studied than MSI2. MSI1 is shown to bind to 3′-untranslated region of its target mRNAs and repress their translational processes [4, 22]. In addition, MSI1 has been found to control the splicing of photoreceptor-specific exons in the retina of vertebrates [41] as well as to regulate the splicing of factors involved in epithelial-luminal state [27]. MSI2 also acts as a translational inhibitor, regulating the function of hematopoietic stem cells [15]. It has also been demonstrated that MSI1 protein regulates memory loss as a part of behavioral plasticity in *C. elegans* [20]. In the past few years, several different RBPs have been identified demonstrating their altered functions and aggregation properties in neurodegenerative diseases [12], among which TDP-43, FUS and TIA-1 are extensively studied (Fig. 1) [11, 40, 47–49]. Abnormal accumulation of tau, a micro-tubule binding protein pathologically characterizes a group of neurodegenerative diseases, known as tauopathies [10]. Tau is believed to bind to RNA and play a role in the quality control of RNA [24, 52, 55]. Moreover, tau interacts with RBPs, such as TIA-1 [3, 51]. More than a decade ago, MSI1 protein was found to be present in tau inclusion-bearing neurons in AD and Pick’s disease (PiD) [36]. Nevertheless, there is no study reporting the involvement of MSI2 protein in neurodegeneration. Co-accumulation of tau oligomers with TIA-1 and other RBPs has been demonstrated in a tauopathy animal model and it has been shown that reduction of TIA-1 levels led to reduced tau oligomers formation, rescuing behavioral deficits in these animals [3, 51]. Another RBP, U1 small nuclear ribonucleoprotein 70 kDa (U1-70 K) protein has also been demonstrated to co-aggregate with tau in both sporadic and familial forms of AD [5].

Herein, we sought to investigate whether the Musashi family members, MSI1 and MSI2 proteins can form aggregates in disease pathology. We observed for the first time in vitro and in ex vivo human AD brains, that these two RBPs are present in their soluble aggregated, i.e., oligomeric forms. These oligomers have been detected in mature neurons that furthermore, co-localize with oligomeric tau. Interestingly, we also observed a change in the patterns of Musashi protein signal, representing cellular distribution of these proteins depending on their association with tau protein. Our observations here suggest that Musashi proteins may have altered cellular localization and possibly dysregulated functions in AD brains, thus contributing to the deleterious effects of aggregated tau.

## Materials and methods

### Preparation of Musashi oligomers

Recombinantly expressed and purified Glutathione-S-transferase (GST)-tagged Musashi proteins. Proteins were then aggregated following our standard protocol [31]. Briefly, both purified MSI1 and MSI2 proteins (0.5 μg/μl) were stirred at 500 RPM with a Teflon-coated micro stir bars for 48 h inside the fume hood at room temperature closed with a cap. MSI1 and MSI2 oligomers were injected into a Shimadzu HPLC system fitted with a TSK-GEL G3000 SWXL (30 cm × 7.8 mm) column, Supelco-808,541. PBS (pH 7.4) was used as the mobile phase with a flow rate of 0.5 mL/min. A gel filtration standard (Bio-Rad 51–1901) was used for calibrations.

### Tissue harvesting

Frontal cortices of frozen brain tissues from AD cases (*N* = 4) and age-matched control subjects (*N* = 4) were received as frozen blocks from the Institute for Brain Aging and Dementia at UC Irvine, approved by the Institutional Ethics Committee. Information about the AD and control cases studied, are summerized in Table 1. Brain tissues (AD, *N* = 4; control, *N* = 4) were homogenized in 1 X PBS mixed with a protease inhibitor cocktail (Roche) and phosphatase inhibitor (Sigma) at 1:3 (*w*/*v*) dilution of brain: PBS. Samples were then centrifuged at 10,000 rpm for 20 min at 4 °C. The supernatants, PBS-soluble fractions were aliquoted, snap-frozen, and stored at − 80 °C until use. The pellets were resuspended in the homogenization buffer (1 X PBS) and were considered as insoluble fractions. They were also aliquoted and frozen at -80 °C until use.

**Table 1:** Brain tissues analyzed in this study from AD and age-matched non-demented control subjects are summarized

Clinical Diagnosis	Age	Gender	PMI (hours)	Braak stage
AD	83	Male	3.5	VI
AD	77	Male	4.5	VI
AD	75	Male	5	VI
AD	75	Female	4.5	VI
Control	79	Female	5	ND
Control	74	Male	3.5	ND
Control	70	Male	5.2	ND
Control	74	Female	3.5	ND

### Immunoprecipitation

Glutathione (GSH) Agarose beads slurry (50%, Pierce) were centrifuged at 1000 x g to discard the storage buffer. Beads were then washed with wash buffer (1X PBS) two times. MSI1 and MSI2 proteins are separately mixed with tau protein at equimolar ratios and incubated with the washed GSH beads on an end-over-end rotator for 4 h at 4 °C. After washing the beads two times with wash buffer, GST-tagged Musashi proteins were eluted with elution buffer (50 mM Tris, 150 mM NaCl, pH 8.0 containing 10 mM reduced glutathione).

### Western blot analysis

Western blot analyses were performed with both recombinant Musashi proteins and homogenates from AD and age-matched control brain tissues. Approximately 4 μg of Musashi oligomeric preparations and 10 μg of each brain homogenate were loaded on precast NuPAGE 4–12% Bis-Tris gels (Invitrogen) for SDS-PAGE analyses. Gels were subsequently transferred onto nitrocellulose membranes and blocked overnight at 4 °C with 10% nonfat dry milk. Membranes were then probed for 1 h at room temperature with α-Oligomeric antibody F11G3 (1:1000), α-MSI1 (1:1000, Abcam), α-MSI2 (1:1000, Abcam) and Tau13 (1:10,000, BioLegend), GAPDH (1:1000, Sigma) antibodies diluted in 5% nonfat dry milk. α-Oligomeric antibody F11G3, α- MSI1 and α- MSI2 immunoreactivity were detected with an HRP-conjugated anti-rabbit IgG (1:6000, GE Healthcare). Tau13 and GAPDH immunoreactivity were detected using an anti-mouse IgG (1:6000, GE Healthcare) diluted in 5% milk. ECL plus (GE Healthcare) was used to visualize the bands.

### Filter trap assay

Approximately 15 μg of brain homogenate from each AD and control case (soluble fractions) was applied onto pre-soaked (TBS-T) nitrocellulose membranes, using a vacuum based bioslot apparatus [17, 35, 53]. After blocking with 10% non-fat dry milk solution, membranes were probed with Tau 13 (1:10,000), α- MSI1 (1:1000) and α- MSI2 (1:1000) antibodies diluted in 5% nonfat milk for 1 h at RT. Immunoreactivity of Tau 13, α-MSI1 and α-MSI2 were detected using an HRP-conjugated anti-mouse IgG (1:6000, GE Healthcare) and an HRP-conjugated anti-rabbit IgG (1:6000, GE Healthcare), respectively.

### Immunofluorescence and confocal microscopy

Immunofluorescence assays were performed with paraffin and frozen sections of frontal cortices from Alzheimer’s disease and control brains. Paraffin sections were deparafinized and rehydrated whereas, frozen sections were fixed in chilled methanol followed by blocking in 5% normal goat serum blocking for 1 h. Sections were then incubated overnight with F11G3 (1:350), Tau5 (1:300, BioLegend), T22 (1:200), α- MSI (1:250), α- MSI2 (1:250) at 4 °C. After washing three times with PBS (10 mins each), sections were then incubated with goat anti-mouse IgG Alexa- 488 and anti-rabbit Alexa 568 (1:700, Thermo Fisher Scientific), as appropriate, for 1 h. They were then washed and mounted with vectashield® mounting medium with DAPI (Vector Laboratories).

### Atomic force microscopy

Oligomeric preparations of both MSI1 and MSI2 proteins were imaged via AFM by ScanAsyst mode with Multimode 8 AFM machine (Bruker, Billerica MA). Briefly, 5 μl of each sample were applied onto a freshly-cleaved mica surface and allowed to adsorb. Mica was then washed with 200 μl of deionized water and air-dried.

### Image analysis

The slides were imaged with an epifluorescence microscope (Nikon Eclipse 800) equipped with a CoolSnap-FX monochrome CCD camera (Photometrics) using standard Nikon GFP, FITC and DAPI filters. Images were processed and analyzed with ImageJ (National Institute of Health, NIH). Confocal microscope (Zeiss LSM 880) has been used to obtain the stacks of brain sections with Nikon 40X objective. Z-Stacks (0.5 μm step size) were used for orthogonal representations. All the colocalization Pearson Correlation Co-effcient (PCC), Scatter plots and Profile plots, presented in this manuscript, have been produced using ImageJ Software (NIH).

### Statistical analysis

All raw data were obtained from Western blot and immunofluorescence quantifications. Statistical analyses of these data were performed, using GraphPad prism 6 software. Integrated densities of quantified signals from Western blot analysis and filter trap assay were plotted as bar graphs, expressed as mean ± standard deviation (SD) for AD and age-matched control brain tissues (*N* = 4, for each group). One-way ANOVA was used to study the occurrence of Musashi proteins in AD cases compared to the control group. To measure the co-localization between Musashi and tau proteins in AD cases, we used Pearson Correlation coefficient (PCC). From each AD brain section, we analyzed 5 ROIs, thus studying 15 ROIs from each case (*n* = 15, ROIs), separately. A representative analysis of such 15 ROIs from a single AD case is provided here. A cut-off value of 0.05 was considered significant for statistical analyses.

## Results

### Formation of recombinant Musashi aggregates in-vitro

**Fig. 2:**
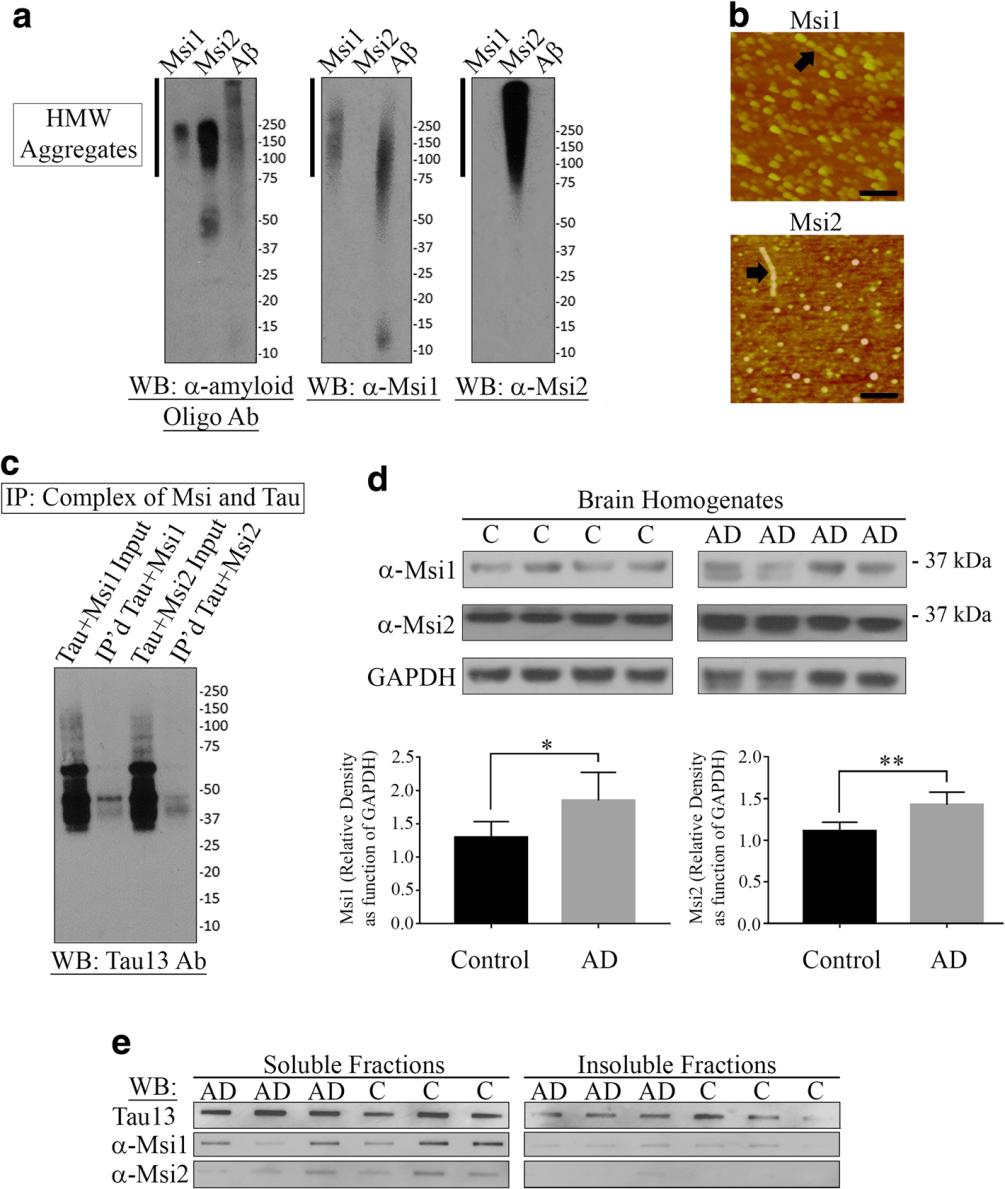
Biochemical and biophysical characterization of recombinant MSI1 and MSI2 protein aggregates (**a**) Western blot images showing in vitro aggregation of recombinant MSI1 and MSI2 aggregates as revealed by immunoblotting with α-Oligomer antibody, F11G3. Generic α-MSI1 antibody detected recombinant MSI1 aggregates, as well as Aβ aggregates. Whereas, α-MSI2 antibody detected only recombinant MSI2 aggregates. (**b**) AFM images of MSI1 and MSI2 protein aggregates showed different morphologies. MSI1 aggregates appeared as pear-shaped oligomers, while MSI2 had mostly spheroid oligomers. Proto-fibrils formation was observed in both MSI1 and MSI2 oligomers (black arrows, black scale bar: 100 nm). (**c**) GST pull down assay of MSI and tau demonstrates their physical interaction. Western blot analysis of immunoprecipitated GST-MSI1 and GST-MSI2 (Lane 2: IP’d GST-MSI1-Tau; Lane 4: IP’d GST-MSI2-tau) with Tau 13 (Pan-tau antibody) revealed the presence of tau proteins in the immunoprecipitated material as well. The inputs from the two reaction mixtures are also shown here (Lane 1: Tau + MSI1 input; Lane 3: Tau + MSI2 input). (**d**) Representative Western blot images of brain homogenates from AD (*N* = 4) and age-matched control cases (*N* = 4) demonstrating a significant increase in the levels of both MSI1 and MSI2 proteins ((*p* = 0.035, *; *p* = 0.005, **). (**e**) Representative images of both soluble and insoluble fractions of AD (*N* = 3) and age-matched control (*N* = 3) brain tissues obtained from filter trap assay. Soluble fractions from all the cases studied demonstrate increased levels of tau protein upon probing with Tau 13 antibody when compared to their insoluble fractions. Similarly, both MSI1 and MSI2 protein levels are increased in the soluble fractions compared to the insoluble fractions (*p* = 0.0279, *; *p* = 0.0045, **)

Western blot analysis of aggregated recombinant Musashi proteins (MSI1 and MSI2) with α-Oligomer antibody, F11G3 [19, 32, 33] detected higher aggregates of the proteins from 75 kDa and above. The aggregates of MSI1 and MSI2 showed higher amount of oligomeric signal around 150–250 kDa. Probing with α-MSI1 antibody we detected MSI1 aggregates. However, this antibody also showed immunoreactivity with Aβ aggregates. Probing with α-MSI2 antibody confirmed the formation of higher aggregates of MSI2 protein starting from ~ 70 kDa and above. This antibody did not show any reactivity with either MSI1 or Aβ aggregates (Fig. 2a). Aggregates of MSI2 higher than 250 kDa were also detected by α-MSI2 antibody which were not detected by the α-Oligomer antibody, suggesting that these could be larger aggregates. Atomic Force Microscopy (AFM) images of both MSI1 and MSI2 aggregates demonstrated their distinctive morphologies. Oligomers of MSI1 protein were pear-shaped, whereas MSI2 were mostly spheroidal in shape. However, proto-fibrillar structures were observed in both the oligomeric proteins (black arrows, Fig. 2b). To investigate if Musashi proteins can directly interact with tau, we performed a Glutathione-S-Transferase (GST) pull-down assay. We incubated separately both MSI1 and MSI2 proteins with tau and glutathione beads and pulled down the possible complexes formed between Musashi proteins and tau. Western blot analysis of immunoprecipitated GST-MSI1 and GST-MSI2 immunolabeled with Tau 13 (a pan tau antibody) revealed the presence of tau proteins in both the immunoprecipitated materials, indicating a possible direct interaction between Musashi proteins and tau (Fig. 2c). Immunolabeling of the GST-MSI and tau complexes with α-MSI1 and α-MSI2 antibodies showed the presence of the corresponding Musashi proteins, thus indicating that both MSI1 and MSI2 were present in the complexes with tau protein (Additional file 1: Figure S1). To investigate the presence MSI1 and MSI2 proteins ex vivo, we have analyzed soluble and insoluble fractions of brain tissues obtained from both AD and age-matched control cases. Western blot analyses of soluble fractions demonstrate significantly increased levels of both MSI1 and MSI2 proteins in AD cases when compared with the controls (*p* = 0.035 *and *p* = 0.005 **, respectively) (Fig. 2d). Furthermore, immunoblots of both soluble and insoluble fractions of AD and control cases obtained from filter trap assay reveal increased levels of MSI1 and MSI2 proteins. However, soluble fractions have a higher level of these two proteins when compared with the insoluble fractions (Fig. 2e).

### Presence of Musashi aggregates in AD brain

**Fig. 3:**
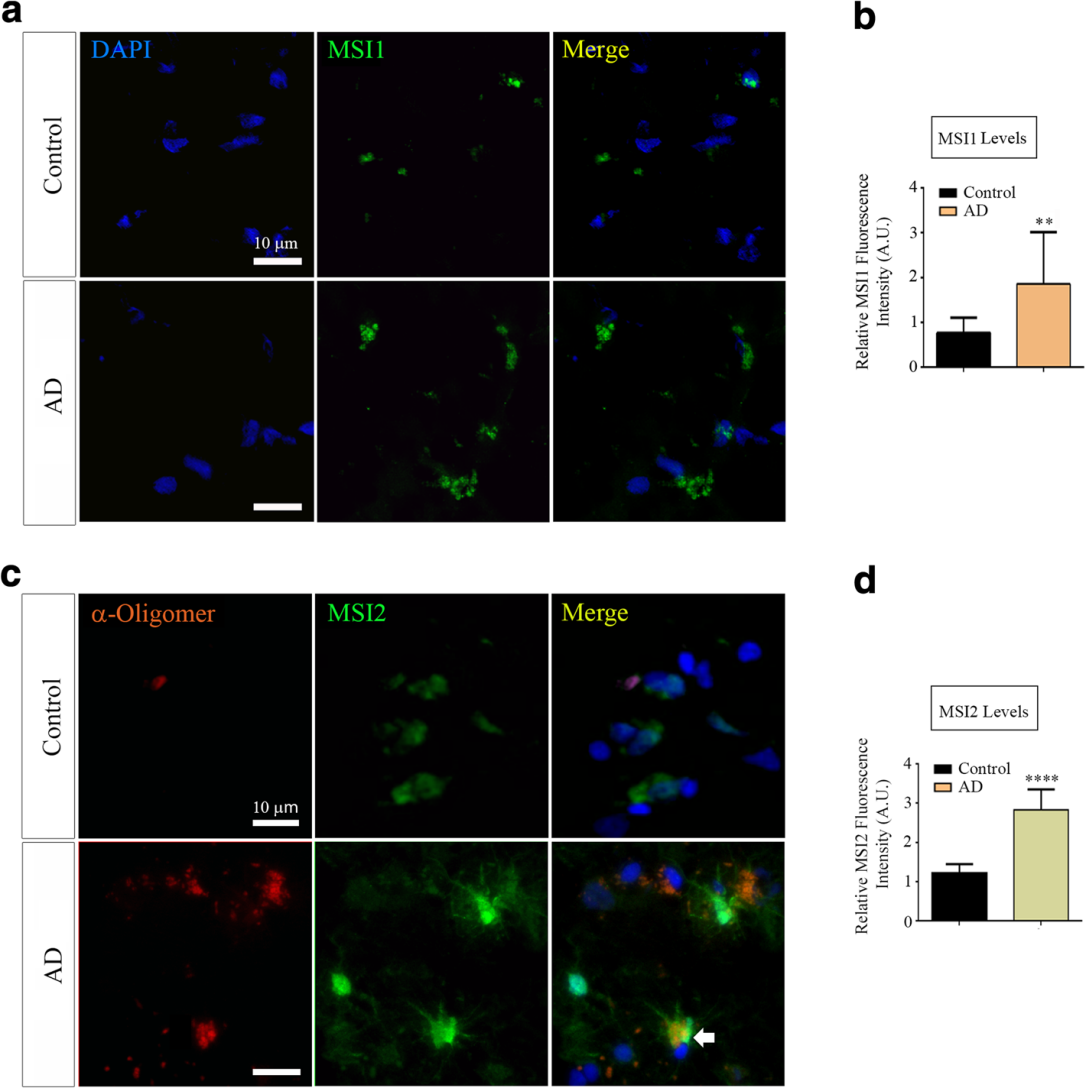
Musashi aggregates in AD (**a**) Representative Epifluorescence images of AD and control cortices immunolabeled with α-MSI1, DAPI (blue, for nuclei) (top) and α-Msi1 (green) and α-Oligomer antibody (F11G3, red) (bottom). An increased MSI1 oligomeric signal was noticed in the cytoplasm of AD brains cortices (white arrows) (**b**) The quantified fluorescence intensity, represented as bar graph demonstrates significantly elevated level of MSI1 signal in AD cortices compared to the controls (*N* = 3 sections per condition*, p* = 0.007, **). (**c**) α-Oligomer antibody (F11G3, red), α-MSI2 antibody (green) and DAPI (nuclei, blue) immunostaining revealed MSI2 oligomers, mostly in the cytoplasm of AD brains cortices (white arrows) (*N* = 3 sections per condition and magnification: 40X, scale bar: 10 μm). (**d**) The fluorescence intensity of MSI2, represented as bar graph reveal significantly elevated levels of MSI2 signal in AD cortex compared to the control (*N* = 3 sections per condition, *p =* 0.0001, ****)

Ex-vivo analysis of AD (cortices) and age-matched non-demented control (cortices) brain tissues were performed by co-immunofluorescence using α-Oligomer antibody (F11G3), α-MSI1 and α-MSI2 antibodies. We observed cytoplasmic localization of MSI1 in AD cortex (white arrow) and control (cortex) tissues (Fig. 3a). Also, AD brains showed higher intensity of MSI1 signal compared to the controls, indicating its elevated levels in AD cortex neurons (Fig. 3b**,** *p =* 0.007, **). Interestingly, we visualized cytoplasmic accumulation of MSI2 oligomers in AD sections (white arrow), but not in the controls (Fig. 3c). The fluorescence intensities of MSI2 signal from AD cortices were measured, plotted and compared to the controls as well. A significant increment of MSI2 signal was detected in AD cortices (*p =* 0.0001, ****) compared to the control (Fig. 3d).

### Distinct cytoplasmic patterns of MSI2 oligomers in AD brain

**Fig. 4:**
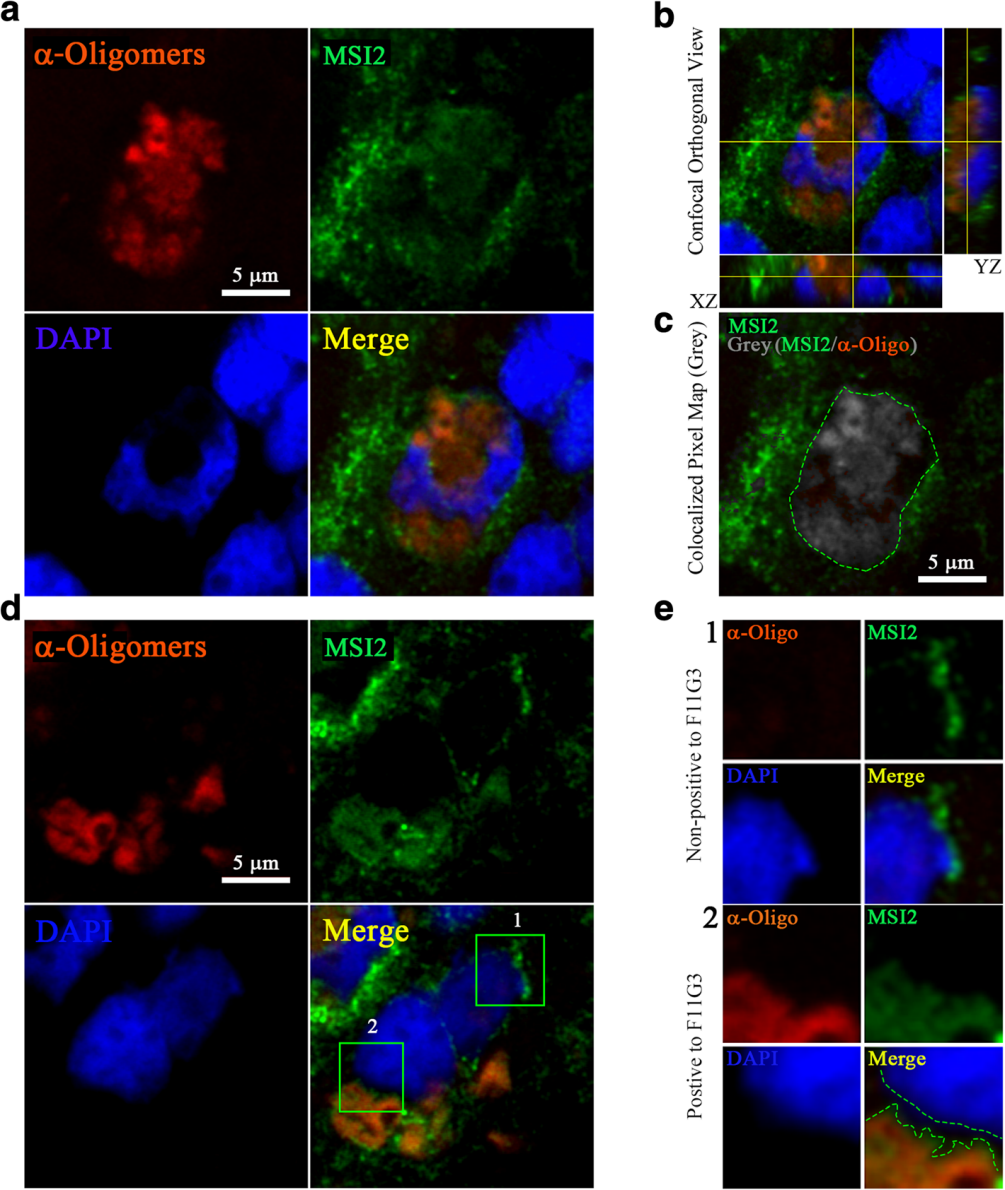
Distinct patterns of MSI2 oligomers in AD brain (**a**) Representative confocal images of AD cortex stained with α-Oligomer antibody (F11G3, red), α-MSI2 antibody (green) and DAPI (blue) for nuclei (white scale bar: 5 μm, magnification: 63X). (**b**) Confocal orthogonal view of image (showed in A) showing the presence of MSI2 oligomers in the perinuclear area. (**c**) Co-localization pixel map between MSI2 (green) and oligomer (red) signals confirmed the overlapping of the two signals (grey area delimitated by green dashed line, white scale bar: 5 μm). (**d**) Confocal (Z-stacks) image demonstrated punctate MSI2 (inset 1) and more diffused distribution of its oligomers (inset 2) in two different cells (white scale bar: 5 μm). (**e-1**) Twice zoomed (inset 1) image showing a proximal localization of punctate MSI2 protein to the nucleus. (**e-2**) Twice zoomed (inset 2) image showing distant localization of MSI2 oligomers from the nucleus (the gap between nucleus and MSI2 oligomers is delimitated by green dashed line)

Confocal imaging revealed oligomeric MSI2 in the cytoplasm of the cell by co-localizing α-Oligomer and α-MSI2 signals (Fig. 4a). Moreover, the orthogonal view demonstrated the perinuclear and partial nuclear distribution of MSI2 oligomers, highlighted by a co-localization pixel map (grey area), the two signals overlapped is delimitated by green dashed line (Fig. 4b, c). More interestingly, MSI2 protein, which was mostly present in punctate distribution and foci, exhibiting a different pattern of signal when it formed oligomers (Fig. 4d). MSI2 had more punctate distribution which was strongly associated with the nuclear membrane (Fig. 4e-1). However, the signal of MSI2 oligomers was mostly diffused and was present in the cytoplasm away from the nuclear membrane (delimitated by green dashed line) (Fig. 4e-2). This observation was assessed in many cells showing that oligomeric forms of MSI2 are present at different grades, following a distinct pattern of distribution in the neurons and with different co-localization patterns (Additional file 2: Figure S2).

### Neuronal MSI2 oligomers in AD brain

**Fig. 5:**
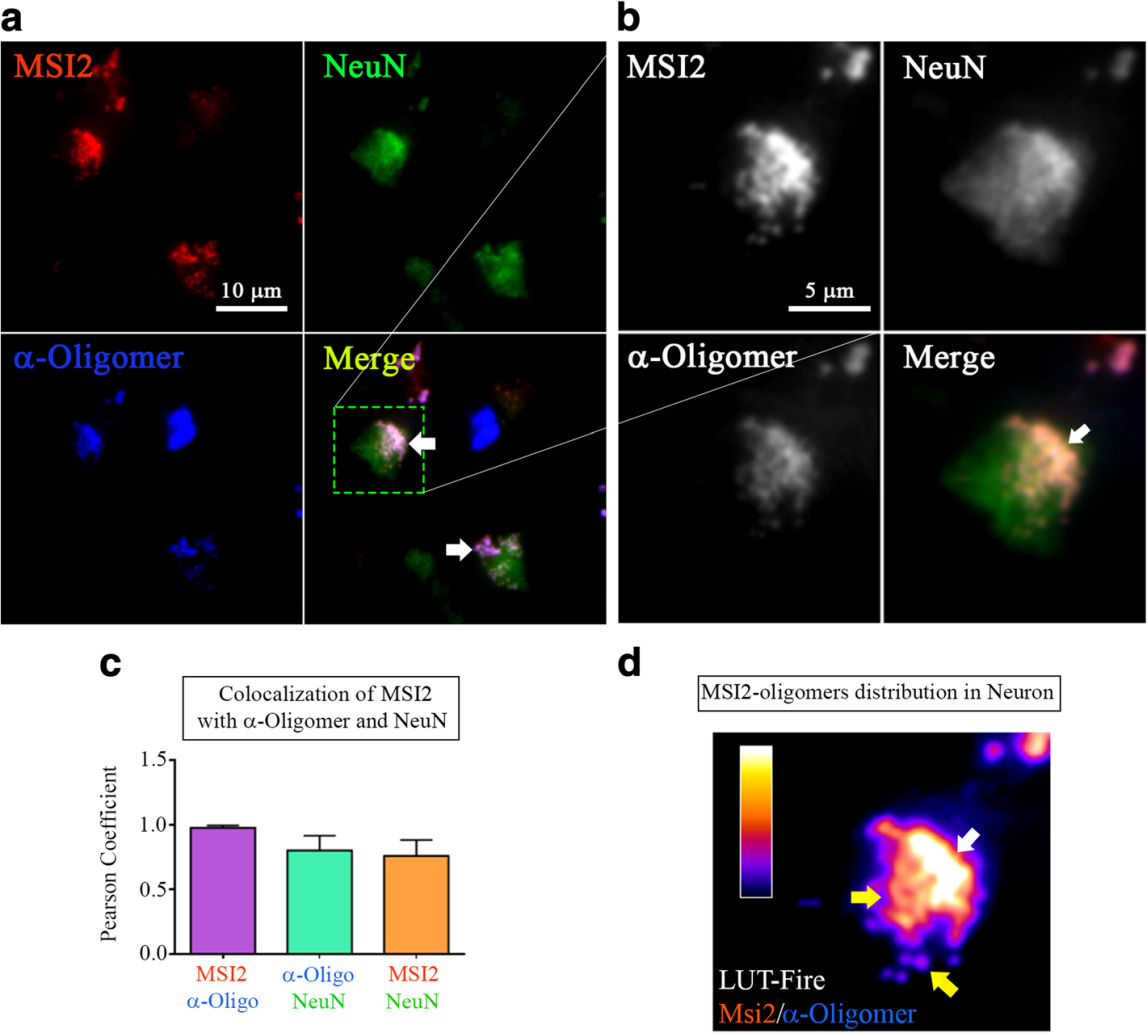
Neuronal MSI2 oligomers in AD Brain (**a**) Representative epifluorescence images of AD cortical section showing co-localization between α-MSI2 (red), NeuN (green) and α-Oligomer antibody (F11G3, blue) (white scale bar: 10 μm, magnification: 40X). (**b**) Twice zoomed image of ROI (green dashed line) highlighting localization of MSI2 oligomers in neurons (white scale bar: 5 μm, magnification 40X). (**c**) PCC of 5 ROIs in AD cortex section represented in bar graphs showing the levels of co-localization between MSI2 and F11G3 (*PCC*: 0.9768 ± 0.01763), MSI2 and NeuN (*PCC*: 0.7581 ± 0.1237) and F11G3 and NeuN (*PCC*: 0.8004 ± 0.1149) signals. (**d**) LUT Fire image of selected ROI between MSI2 and α-Oligomer antibody (F11G3) confirming the presence of MSI2 oligomers in neurons with heterogeneous distribution in the cytoplasm (color scale bar represents pixel intensity)

To investigate neuronal localization of MSI2 oligomers, we performed triple-staining of AD brain cortical sections with NeuN (neuronal marker), α-Oligomer antibody (F11G3) and α-MSI2 antibodies. We observed MSI2 oligomers in the cytoplasm of neurons (white arrows) by triple co-localization between F11G3, α-MSI2 and NeuN signals (Fig. 5a). A twice zoomed image (dashed green square in the merge) demonstrated co-localization between NeuN and MSI2 oligomers in the cytoplasm (white arrow), indicating that these oligomers were present in mature neurons (Fig. 5b). To further confirm the co-localization of the signals, we performed a Pearson Correlation Coefficient analysis (PCC) of the regions of interests (ROIs), represented as bar plots. We observed a strong association between MSI2 and F11G3 (*PCC*: 0.9768 ± 0.01763). Pearson Correlation Coefficients were also calculated for MSI2 and NeuN (*PCC*: 0.7581 ± 0.1237) and NeuN and F11G3 (*PCC*: 0.8004 ± 0.1149). All these data suggest that MSI2 oligomers are mostly localized in neuronal cytoplasm (Fig. 5c). To define the distribution of MSI2 oligomers in the neurons, the intensities of the co-localized channel (MSI2/F11G3) were converted in a Lookup Table (LUT - Type Fire) (Fig. 5d). We observed a less homogenous distribution of oligomers in some areas rich in aggregates (white arrows) and other areas with spotted and diffused signals (yellow arrows).

### Co-localization of Musashi proteins with tau and its oligomeric forms in AD brain

**Fig. 6:**
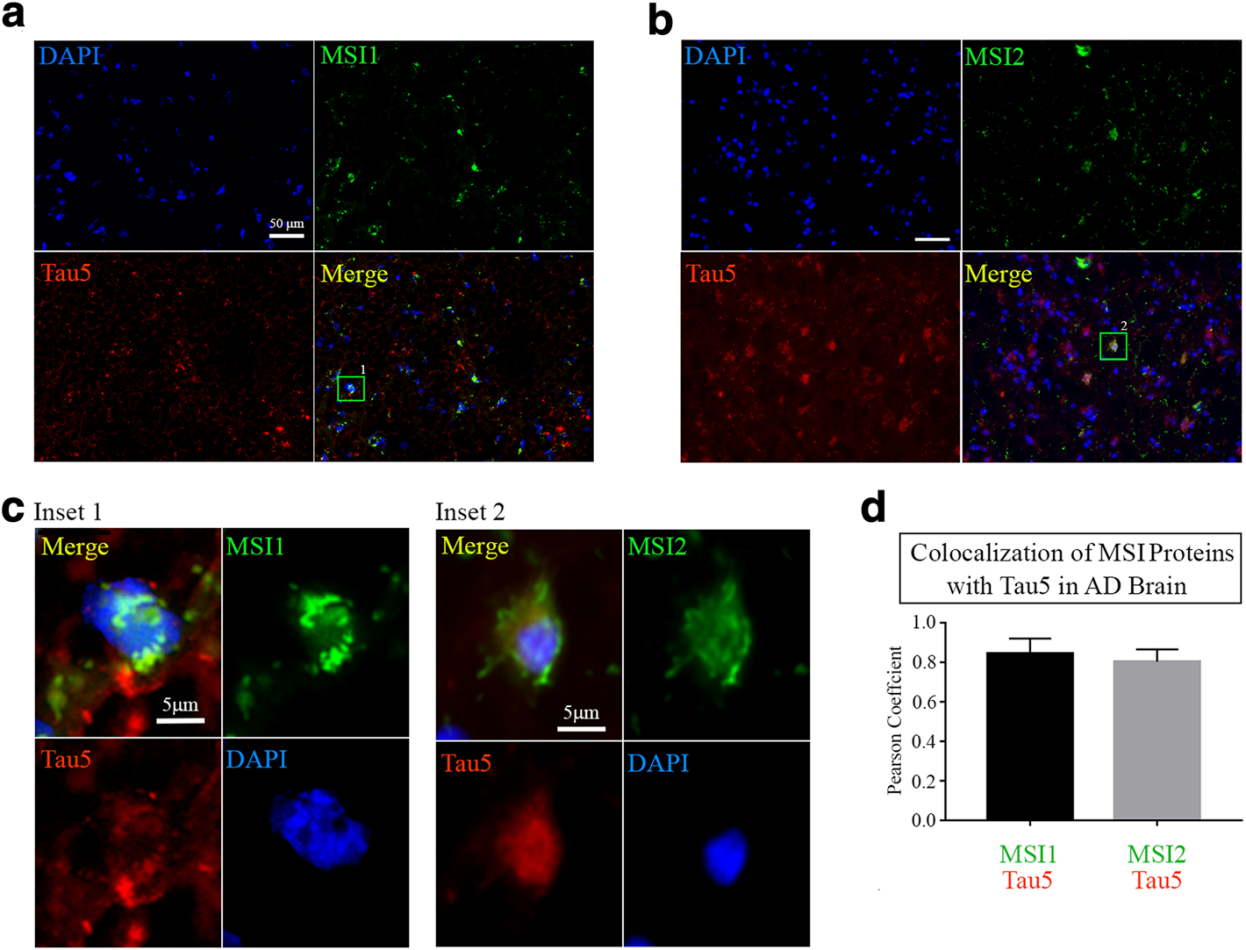
Co-localization of MSI1 and MSI2 with tau in AD Brains (**a**) Representative epifluorescence image of AD cortex section stained with α-MSI1 and Pan-tau (Tau5) antibodies (white scale bar: 50 μm, magnification: 10X). (**b**) Representative epifluorescence image of AD cortex section stained with α-MSI2 and Pan-tau (Tau5) antibodies (white scale bar: 50 μm, magnification: 10X). (**c**) Inset 1: ten times zoomed image (green square in **a**) showed diffuse cytoplasmic co-localization of MSI1 and tau (white scale bar: 5 μm). Inset 2: ten times zoomed image (green square in **c**) showed cytoplasmic co-localization of MSI2 and tau (white scale bar: 5 μm). (**d**) PCC Graph represent colocalization coefficient between MSI1/Tau (*PCC*: 0.84 ± 0.07) and MSI2/Tau (*PCC*: 0.80 ± 0.06) in positive cells to both proteins showing high association in AD brain

**Fig. 7:**
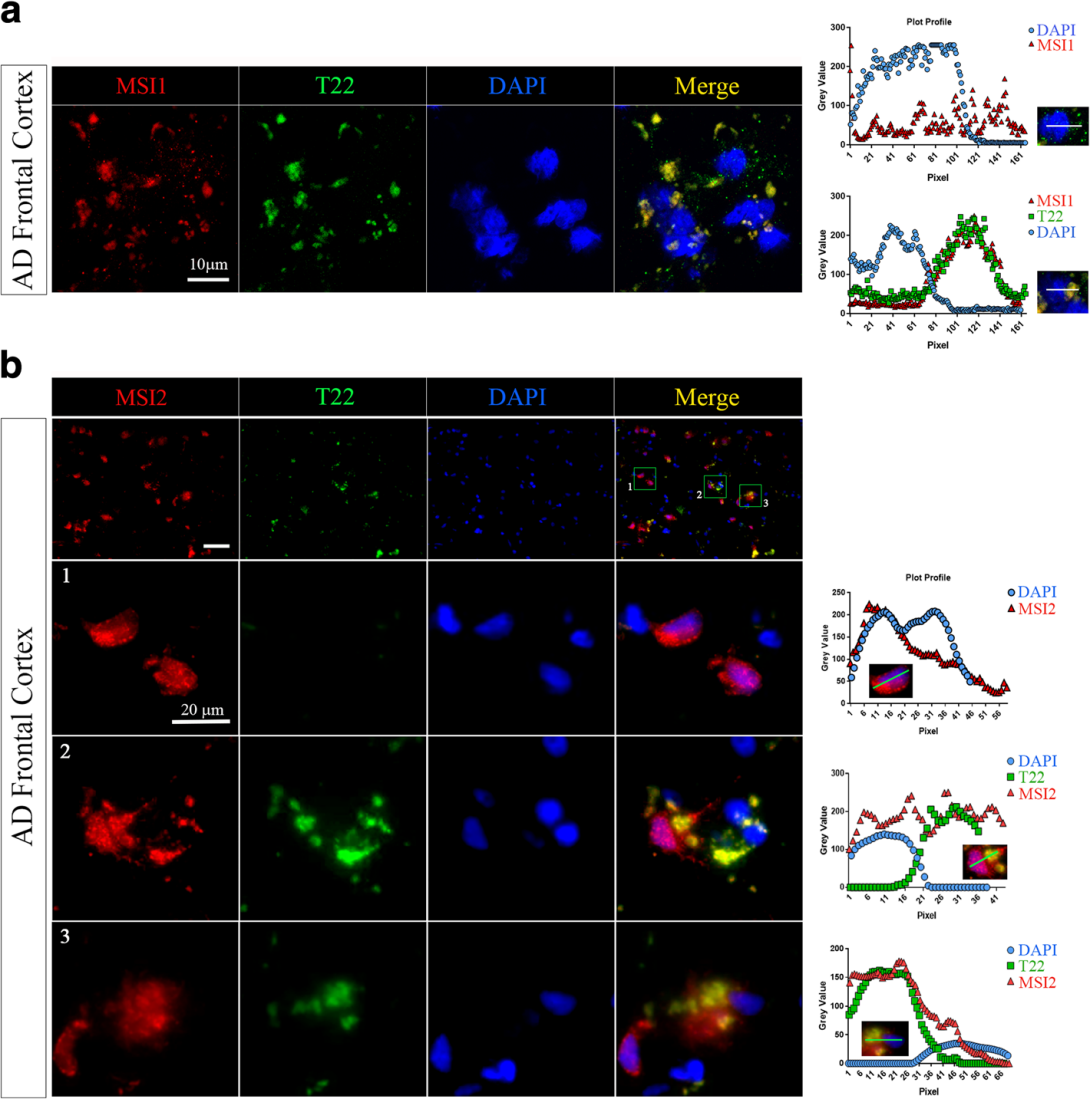
Co-localization of MSI1 and MSI2 with tau oligomers in AD Brain (**a**) Epifluorescence images of AD cortex section stained with α-MSI2 (red) and α-tau oligomeric antibody, T22 (green), DAPI (Nuclei, blue). Co-localization was observed in large portion of the tissue. Different distribution pattern was observed. Area positive to MSI1 spot distribution and negative signal of T22 (top graph) and strong co-localization was observed in same cells between MSI1 and T22 in the cytoplasm (bottom graph). The fluorescence intensity was measured and represented as intensity plot profile (DAPI, blue; MSI2, red and TauO, green). These signals have been observed in different sections of cortices (white scale bar: 10 μm magnification, 20X zoomed twice). (**b**) Epifluorescence images of AD cortex section stained with α-MSI2 (red) and α-tau oligomeric antibody, T22 (green). Different Co-localization grades were evaluated in 3 different ROIs (green squares) (white scale bar: 50 μm, magnification: 40X). Image 1 (MSI2+/TauO-), Image 2 (MSI2+/TauO+) and Image 3 (MSI2+/TauO+) showed different distribution patterns of MSI2 and tau oligomers (white scale bar: 20 μm, magnification: 40X). One ROI from each image (1–3) was selected and the fluorescence intensity was measured which is represented as intensity plot profile (DAPI, blue; MSI2, red and TauO, green)

To investigate the association between MSI1 and MSI2 with tau protein, we performed co-staining of cortical human AD sections with α-MSI1 α-MSI2 antibodies and Tau5 (Pan-tau antibody). We observed positive cells for MSI1 and Tau (Inset 1, green square) as well as cells positive to MSI2 and Tau (Fig. 6a, b; green squares). The distribution of Musashi proteins has been observed to be not homogenous along the cells in the cortex sections. With enhanced magnification, we observed mostly cytoplasmic localization of MSI1 (Fig. 6c, Inset 1) and MSI2 (Fig. 6c, Inset 2) and their association with tau protein in the cells. We also evaluated co-localization with PCC analysis between MSI1/Tau5 and MSI2/Tau5 signals showing a strong association (MSI1/Tau5 *PCC*: 0.8454 ± 0.0762 and MSI2/Tau5 PCC: 0.8036 ± 0.06294, Fig. 6d). Immunostaining of AD sections with α-MSI1 and α-MSI2 and α-tau oligomeric antibody (T22) showed strong co-localization of MSI1 and MSI2 with tau oligomers in AD cortex. We observed cells positive to both MSI1 and T22 signal in the cytoplasm (Fig. 7a) with differential pattern of MSI1 and T22 expressed by intensity plots. Further analyses of different ROIs (1–3 green squares) revealed heterogeneous distribution of MSI2, particularly, in relation to tau oligomers (TauO) presence (Fig. 7a**)**. In detail, Image 1 shows MSI2+/TauO- cells with a strong nuclear and perinuclear presence of MSI2, as shown by intensity profile plot. In image 2 (MSI2+/TauO+), we observed a co-localization of MSI2 with tau oligomers in the cytoplasm. Furthermore, we observed a concomitant increment of distance of MSI2/TauO aggregates from the nucleus. In image 3 (MSI2+/TauO+), we observed a cloudy distribution of co-localized MSI2 and tau oligomers which was different from Image 2 where we mostly observed a compact distribution. For all ROIs selected, plot profiles were generated indicating intensity distribution of co-localized regions in the cells selected (Fig. 7b).

## Discussion

Over the past decades, RBPs, their dysregulation and toxic roles in neurodegenerative diseases are being actively investigated. Aggregation of many RBPs, such as TIA-1 [18], FUS, TDP43, hnRNPA1 and hnRNPA2 in Amyotrophic lateral sclerosis/frontotemporal dementia (ALS/FTD) proteinopathies are mediated by prion-related domain (PRD) [29, 39]. RBPs also possess RNA-Recognition Motifs (RRMs) by which they interact with RNA molecules. These motifs are found to be conserved for each protein. Apart from such conserved RRMs, the RBPs also possess a glycine-rich domain that is also conserved. This glycine-rich domain is hydrophobic in nature, allowing the reversible aggregation of these proteins as shown with FUS and TDP-43. These two RBPs are strongly implicated in neurodegenerative diseases, such as ALS and AD [16, 23]. Although they are nuclear proteins, cytoplasmic localization of these proteins is noted in stress granules containing aggregates. Apart from AD, the pathological inclusions of tau protein also characterize a group of neurodegenerative diseases, collectively known as tauopathies [10]. Interactions between tau and other RBPs have been demonstrated in neurodegenerative diseases. The Musashi proteins, another group of RBPs are mostly studied to play roles during neurogenesis [26]. There is only one study that had demonstrated the existence of MSI1 protein in neurons bearing tau inclusions in AD and PiD pathologies [36]. However, the occurrence of MSI2 protein and their toxic form of aggregation, i.e., oligomers have not been investigated yet in neurodegeneration. To the best of our knowledge, this is the first study demonstrating Musashi proteins’ aggregation state, specifically the oligomers in AD pathophysiology and their co-occurrence with tau oligomers. We have demonstrated that recombinant MSI1 and MSI2 proteins can be aggregated in vitro as shown for other amyloidogenic proteins, following our published protocol [31].

**Fig. 8:**
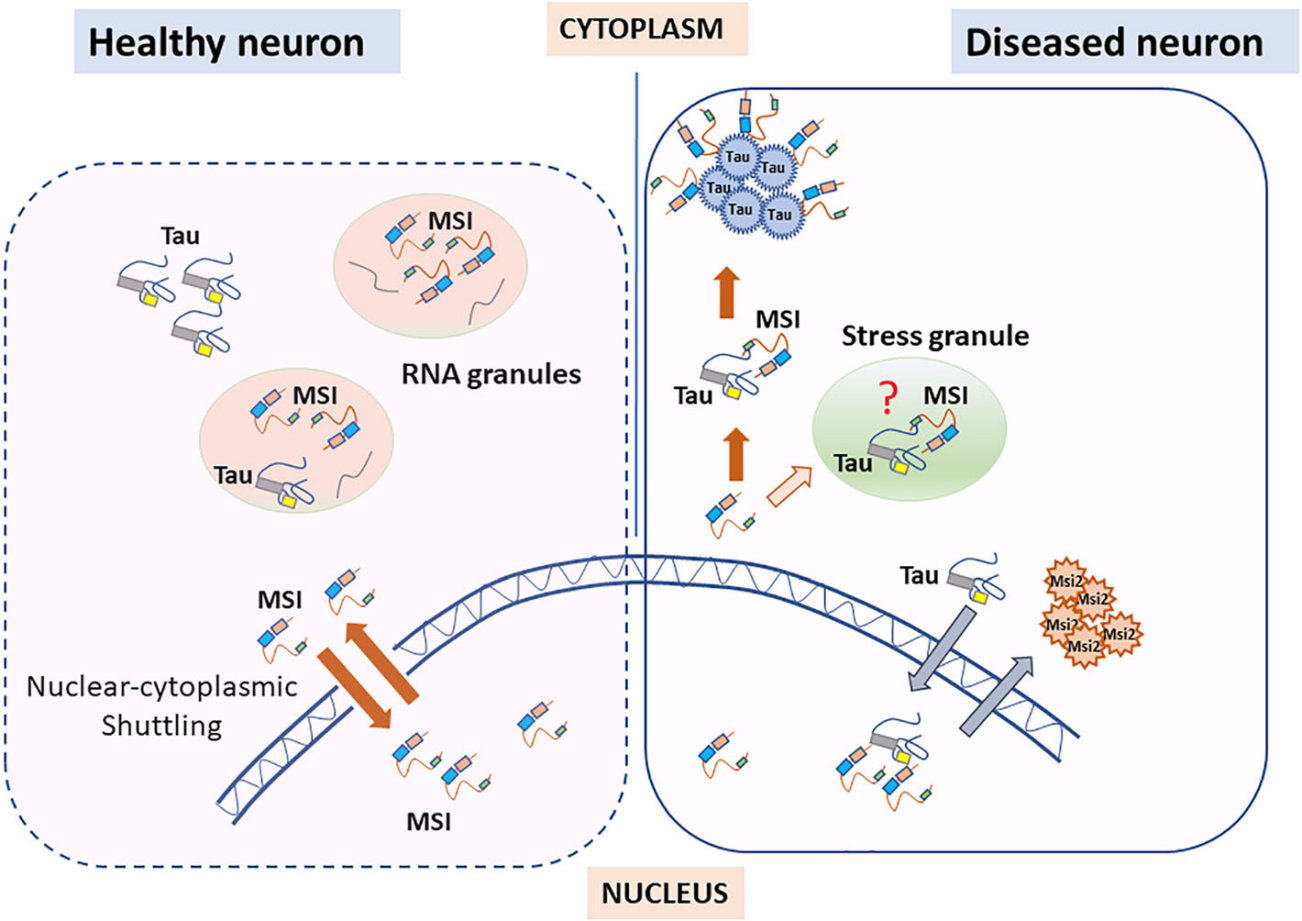
Hypothetical model of Musashi protein aggregation and its interaction with tau oligomers in AD pathology. The dotted area (left) depicts nuclear-cytoplasmic shuttling of Musashi proteins and their localization in RNA granules based on the published literatures. Additionally, tau protein has also been reported to be involved in RNA granule trafficking. The area under solid line (right) depicts the observations made in this study. Musashi proteins co-localized with tau protein, as well as its oligomeric forms in the cytoplasm. In diseased state, these two proteins might be present in the stress granules. Moreover, mislocalization of tau protein in the nucleus and its subsequent interaction with Musashi proteins may lead to impaired functioning of Musashi proteins, thus resulting in its aggregation

It is suggested that proteins present in supersaturated concentration in the cellular environment are driven to form aggregates [9]. In our study, we have observed an elevated level of MSI1 and MSI2 protein in AD brain tissues compared to the age-matched controls. Musashi proteins are predominantly present in the cytoplasm with spot distribution but are also expressed in the nucleus [37]. The sub-cellular localization of these proteins is also supported by the presence of nuclear localization and nuclear export signals. We propose two possible hypotheses by which the Musashi oligomers could exist in AD pathology and interact with tau oligomers (Fig. 8). It is known that Musashi proteins shuttle between nucleus and cytoplasm. Therefore, MSI2 might interact with tau in the cytoplasm as observed here and this may drive tau into the nucleus. We consider this pathway as “cytoplasmic hypothesis”. In AD pathology, such interaction eventually might lead to the aggregation of tau forming tau oligomers co-localizing with MSI2 protein in the cytoplasm. Proteins that are intrinsically disordered and have low complexity regions are shown to drive Liquid-Liquid Phase Separation (LLPS) and form stress granules. This phenomenon has been suggested as a compelling mechanism for the aggregation of such disordered proteins in the granules and thus, causing toxicity [38]. Tau is also reported to undergo such phase separation forming tau droplets [2]. However, whether such interaction is occurring in the stress granules or other RNA granules while carrying RNA transcripts has not been investigated in this study. Studies have highlighted the role of RBPs in toxic tau aggregation in the disease pathology. Tau is shown to bind with TIA-1, another core stress granule RBP leading to its cytoplasmic localization and aggregation. Such interaction modulates the interaction of tau with other RBPs [3, 51]. As a second hypothesis, sub-cellular localization of tau protein might also play role in Musashi protein aggregation. Though, tau is a microtubule binding protein and primarily present in the cytoplasm, the nuclear localization of tau has also been suggested to play role in AD pathology [6, 8]. Alternately, tau protein could mis-localize into the nucleus and interact with nuclear MSI1 and MSI2. Such mis-localization of tau can cause the aggregation of Musashi proteins and hinder their normal functionality, as MSI2 aggregates were observed in the cytoplasm away from the nucleus. We consider this pathway as “nuclear hypothesis”.

**Fig. 9:**
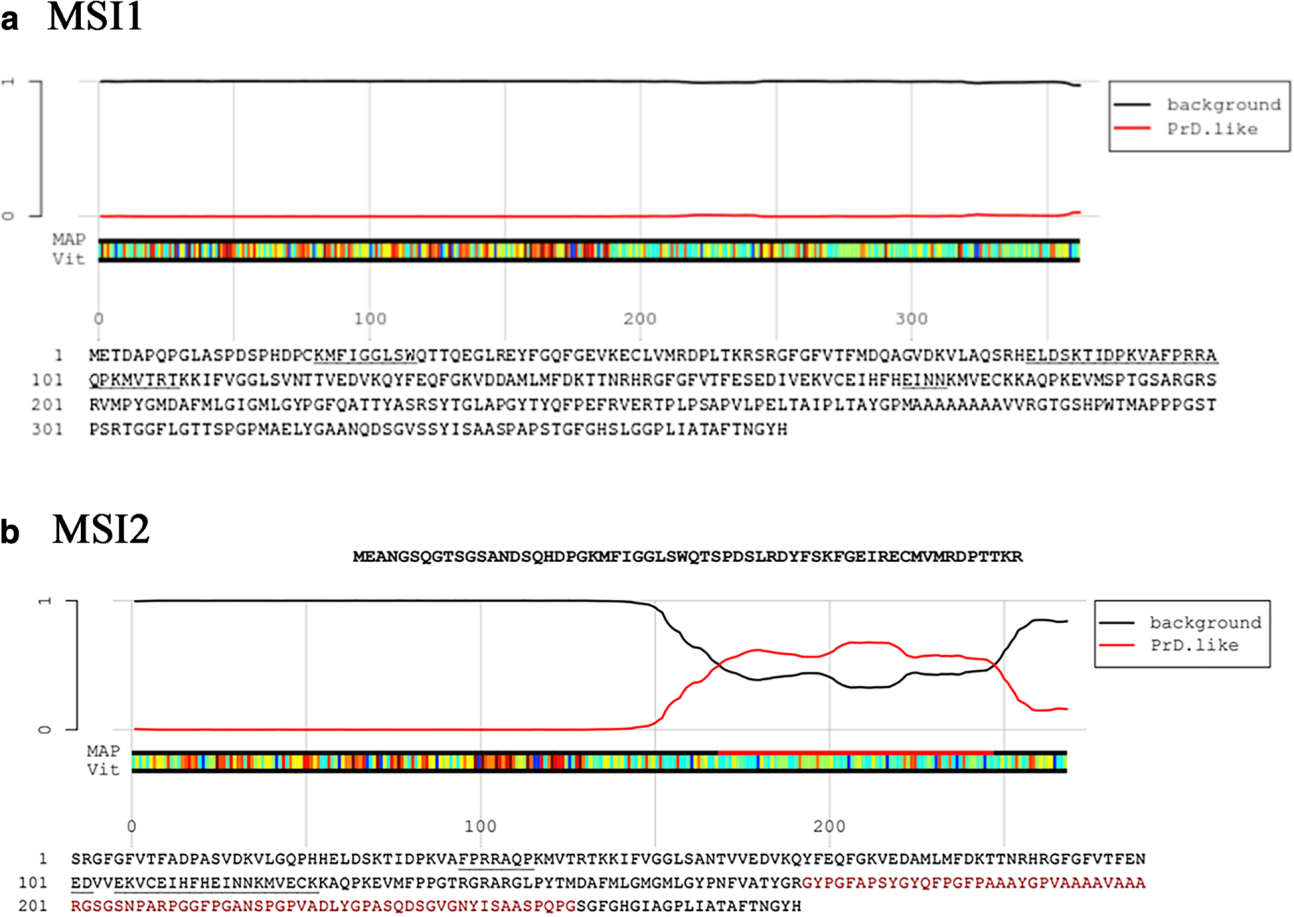
Analyses of Musashi proteins by PLAAC software to identify prion-like region. **a** MSI1 protein does not show the presence of prion-like domain analyzed by this algorithm, whereas (**b**) MSI2 protein reveals the occurrence of such domain, as indicated by the red line and residues in red

Tau is observed to be present in the RNA granules formed under stress, known as stress granules [7]. In a healthy state, Musashi proteins can be found in polysomes [28], and being shuttled in and out of the nucleus. We observed the accumulation of Musashi proteins in the cytoplasm and nucleus, thus indicating their altered functions, which could be attributed to the mis-localization of tau into nucleus. Alternatively, under stress condition, Musashi proteins might interact with tau in the cytoplasm. Interaction of Musashi proteins with tau might initiate the aggregation of the Musashi proteins (Fig. 8), as observed for TIA-1 protein. The role of Musashi proteins’ structures influencing their aggregation process` in neurodegeneration is not yet elucidated. Thus far, there is no study reporting the occurrence of any PRD or low complexity domain (LCD) in Musashi proteins that would indicate their aggregation propensity. Here in, we have attempted to address our observation of Musashi oligomer formation in vitro and as well as in AD brain tissues by analyzing their sequences. To identify if there is any Prion-like domain present in Musashi proteins or not, we analyzed the amino acid sequences of MSI1 and MSI2 proteins in Prion-Like Amino Acid Composition (PLAAC) software [1, 13]. The output results reveal that a stretch of 169–247 amino acid residues in MSI2 protein might have prion-like properties which can cause the aggregation of this protein (Fig. 9). However, MSI1 protein sequence did not show the presence of any such prion-like domain from the PLAAC analysis. Such observation requires further studies to confirm the presence of a prion-like region and its role in the aggregation process. Taken together, our findings suggest that Musashi proteins can form soluble amyloid aggregates and may have dysregulated functions in AD brains, thus contributing to the deleterious effects of tau protein aggregation. This study provides a novel insight in the complexity of protein aggregation in neurodegeneration and demonstrates the role of a significant but less studied group of RBPs, the Musashi proteins in brain diseases.

## Additional files

Additional file 1: **Figure S1.** Biochemical characterization of recombinant MSI1 and MSI2 proteins forming complexes with tau protein, separately. (TIF 14200 kb)

Additional file 2: **Figure S2.** Distinct pattern of cytoplasmmic accumulation of MSI2 protein in AD brain tissues. (TIF 3070 kb)
