# Supplementary figures and images for: Formation of Toxic Oligomeric Assemblies of RNA-binding Protein: Musashi in Alzheimer’s disease
Source: Acta Neuropathol Commun. 2018 Oct 26;6:113. doi: 10.1186/s40478-018-0615-0 (PMC6203984; doi:10.1186/s40478-018-0615-0)

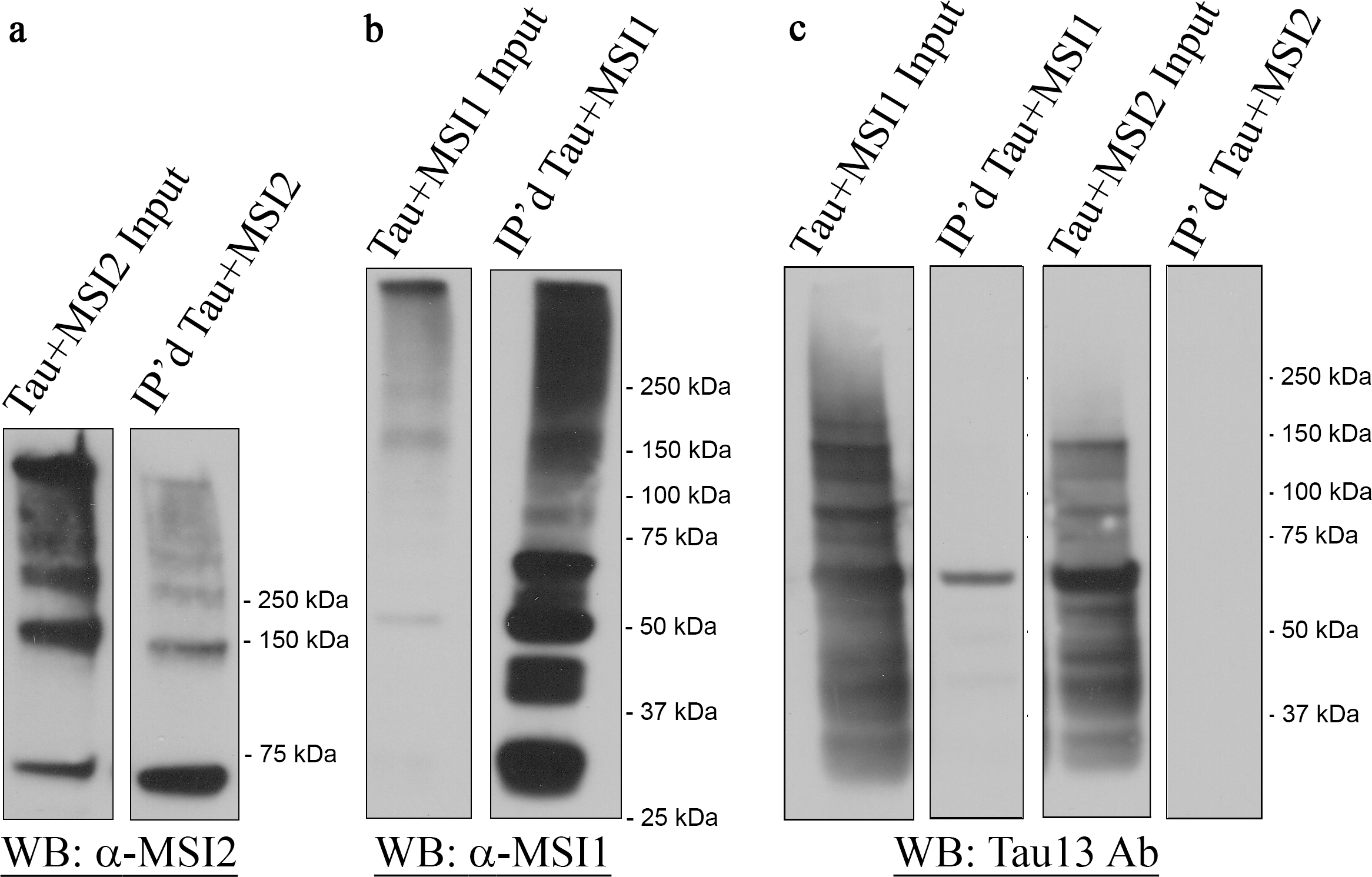

Supplement: Supplementary file 1 — Figure S1. Biochemical characterization of recombinant MSI1 and MSI2 proteins forming complexes with tau protein, separately. (TIF 14200 kb) [file 40478_2018_615_MOESM1_ESM.tif]

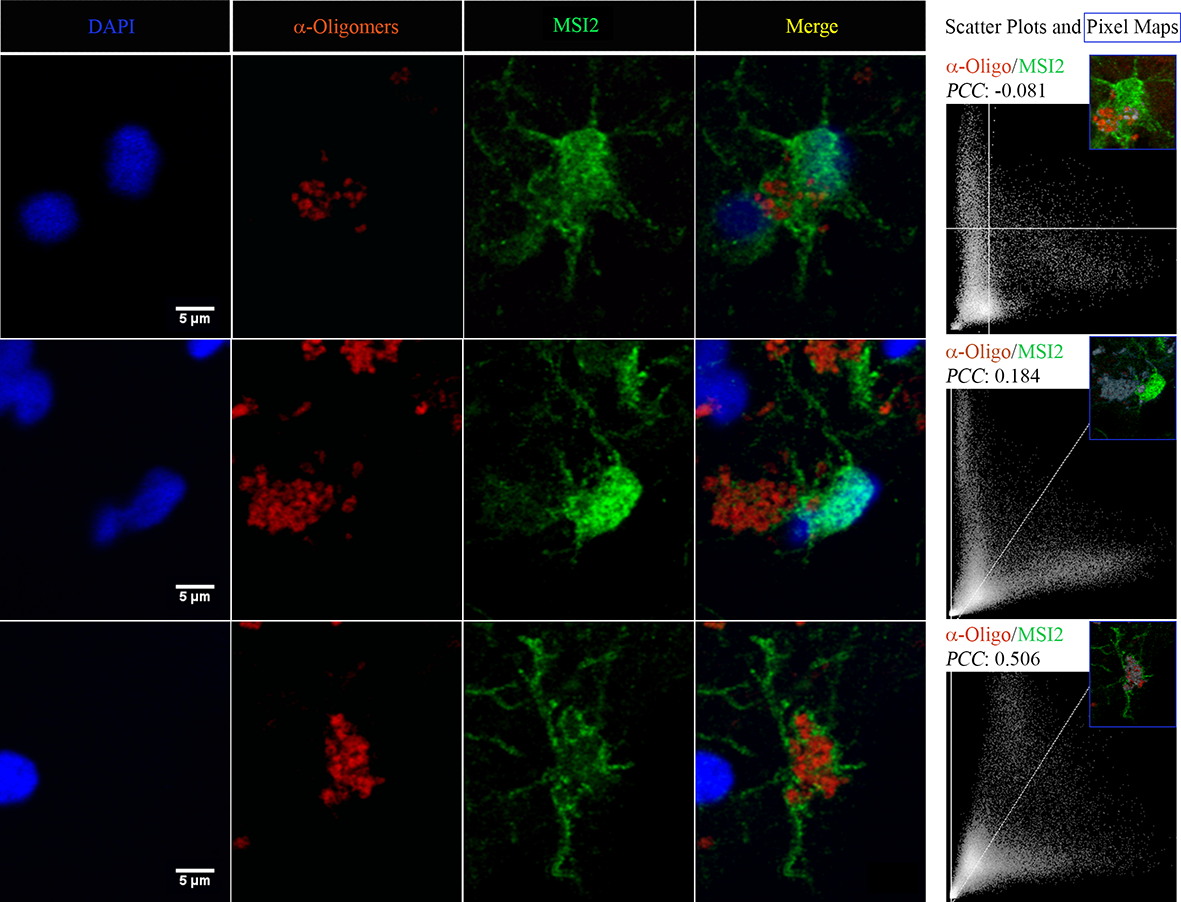

Supplement: Supplementary file 2 — Figure S2. Distinct pattern of cytoplasmmic accumulation of MSI2 protein in AD brain tissues. (TIF 3070 kb) [file 40478_2018_615_MOESM2_ESM.tif]
